# Ocular surface parameter changes in the untreated fellow eye after unilateral cataract surgery with short-term administration of anti-inflammatory eye drops

**DOI:** 10.1038/s41598-024-51764-7

**Published:** 2024-01-11

**Authors:** Seung Hyeun Lee, Yeoun Sook Chun, Kyoung Woo Kim

**Affiliations:** Department of Ophthalmology, Chung-Ang University College of Medicine, Chung-Ang University Hospital, 102 Heukseok-ro, Dongjak-gu, Seoul, 06973 Republic of Korea

**Keywords:** Health care, Eye diseases, Lens diseases

## Abstract

This study aimed to investigate the changes in clinical parameters of dry eye disease and meibomian gland dysfunction in both the operated and untreated fellow eyes of patients who underwent unilateral cataract surgery with the short-term administration of anti-inflammatory eye drops in the surgical eye. The medical charts of 57 consecutive patients who underwent unilateral cataract surgery and received 1% prednisolone acetate and non-steroidal anti-inflammatory drug (NSAID, 0.1% bromfenac sodium) eye drops were reviewed. The preoperative ocular surface disease index questionnaire score (38.9 ± 20.5) decreased significantly to 15.2 ± 16.4 at post-surgical 1 week and further to 12.8 ± 11.4 after 1 month. Although meibum quality grade increased and corneal sensitivity decreased at 1 week in operated eyes, corneal erosion scores and Sjogren’s International Collaborative Clinical Alliance ocular staining scores even improved over a month in the untreated fellow eyes. The tear matrix metalloproteinase (MMP)-9 grade decreased in both operated eyes and untreated fellow eyes after 1 month from surgery. In conclusion, the short-term topical anti-inflammatory treatment using steroid and NSAID eye drops in the operated eye after cataract surgery decreased subjective ocular surface discomfort and improved ocular surface staining scores and tear MMP-9 expression in the untreated fellow eyes.

## Introduction

Dry eye disease (DED) is a multifactorial condition that disrupts the stability of the ocular surface, tear film, and sensory nerves, leading to increased tear osmolarity and discomfort^1,2^. While the traditional belief was that DED is primarily caused by tear film disturbances, recent consensus has highlighted the role of neurogenic stress and ocular surface inflammation^3,4^.

After cataract surgery, DED is one of the most common discomforts reported by patients. This condition can occur due to the surgery or may worsen preexisting dry eye^5,6^. According to a meta-analysis of DED prevalence after cataract surgery, overall 37.4% of patients without preexisting DED developed DED following the cataract surgery^7^. Several factors contribute to this condition^8^, such as corneal nerve injury^9,10^, povidone iodine^11^, mechanical stimulation and inflammation by the use of lid speculums during surgery^12^, and prolonged exposure to intense light from a microscope^13^. Additionally, the effects of benzalkonium chloride in topical anesthetic agents and post-surgically used eye drops may be related^14,15^. Amongst, a full-thickness corneal incision during surgery is one of the strongest contributors to the development of DED after cataract surgery^16^, leading to decreased tear production and tear film instability^17,18^. Inflammatory mediators induced by surgically induced neurogenic inflammation can sensitize corneal polymodal nerves^19^, which may cause postsurgical persistent pain^20^. To prevent or relieve such a mechanism, postsurgical corticosteroid and nonsteroidal anti-inflammatory drug (NSAID) use has been effective^20^.

The corneal nerve plays a critical role in maintaining a healthy ocular surface and regulating immune responses^21,22^. Damage to the corneal nerves can induce neurogenic inflammation by releasing inflammatory neuropeptides, such as substance P^19^. It is interesting to note that damage to the corneal nerves on one side can also affect immune cell activity, corneal nerve density, and cytokine levels in both the affected and unaffected eyes^23–25^. In a mouse model, severing the unilateral corneal nerve led to immune cell activation, dysregulated lacrimal secretion, and increased corneal staining score bilaterally through bidirectional neuronal and immunogenic signals^26^. Additionally, a circular corneal incision in one eye can abolish the immune privilege of the bilateral ocular surface, leading to a higher rate of corneal allograft rejection^27^.

The objective of a study conducted on a cohort of patients who underwent unilateral cataract surgery was to investigate short-term changes in clinical indicators of DED and meibomian gland dysfunction (MGD) in both the untreated fellow eye and the operated eye, with the short-term administration of anti-inflammatory eye drops in surgical eyes.

## Results

### Demographics and baseline parameters

A total of 57 subjects participated in this study with a mean age of 70.4 ± 9.7 years. Females made up 43.9% of the cohort (Table 1). The severity of MGD and all parameters of DED did not differ significantly between the groups (Table 2).

**Table 1:** Demographics of subjects enrolled in this study.

Variables	Value
Total no. of patients	57
Age	70.4 ± 9.7
Sex (% female)	43.9%

**Table 2:** The baseline values of the clinical parameters of the ocular surface measured in both the operated eyes and the untreated fellow eyes prior to the cataract surgery.

Parameters	Group		*P* value
	Operated eye	Fellow eye	
MG expressibility (Gr)^a^	1.72 ± 0.54	1.60 ± 0.54	0.300
Meibum quality (Gr)^b^	1.59 ± 0.80	1.63 ± 0.71	0.784
Schirmer I without anesthesia (mm)^a^	12.4 ± 8.8	11.1 ± 7.0	0.698
Corneal sensitivity (cm)^a^	5.8 ± 90.35	5.79 ± 0.61	0.768
Tear BUT (sec)^b^	6.82 ± 2.54	6.54 ± 2.44	0.670
Corneal erosion score (NEI)^a^	1.11 ± 1.53	0.93 ± 1.36	0.675
OSS score (SICCA)^a^	0.63 ± 1.01	0.56 ± 1.01	0.688
Tear osmolarity (mOsm/L)^b^	315.7 ± 22.9	317.1 ± 19.4	0.809
Tear MMP-9 (Gr)^b^	1.93 ± 0.98	1.93 ± 1.16	1.000

*MG* meibomian gland, *Gr* grade, *BUT* break-up time, *NEI* National Eye Institute/Industry, *OSS* ocular staining score, *SICCA* Sjogren’s International Collaborative Clinical Alliance, *MMP* matrix metalloproteinase.

^a^Mann-Whitney U test.

^b^Student’s t-test.

### Change of ocular surface disease index (OSDI) questionnaire scores

**Figure 1:**
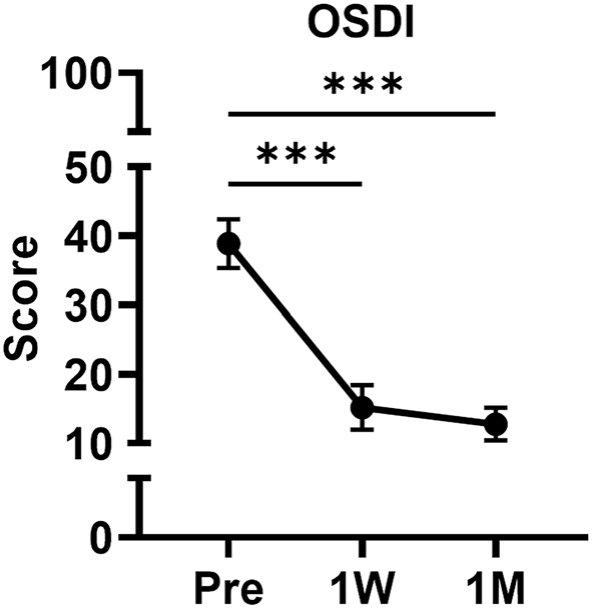
The short-term time-serial change of OSDI score before and after cataract surgery for 1 month. The OSDI score was significantly decreased at both 1 week and 1 month compared to the baseline. ***P < 0.001 (One-way repeated measure analysis of variance analysis followed by Bonferroni’s *post-hoc*). OSDI, ocular surface disease index. *W* week. *M* month.

The mean baseline OSDI score was 38.9 ± 20.5. This decreased to 15.2 ± 16.4 at 1 week post-surgery and further to 12.8 ± 11.4 after a month (Fig. 1). Both changes at postoperative 1 week and 1 month compared to baseline were statistically significant (*P* < 0.001 and *P* < 0.001, respectively). The OSDI scores did not correlate significantly with any of the DED parameters and MGD severity grades, both from the operated eyes and untreated fellow eyes, before cataract surgery as well as 1 month after surgery.

### Change in DED parameters and MGD severity grades

According to repeated measure analysis of variance (RMANOVA) analysis, meibum quality grade, tear secretion by Schirmer I test, and corneal sensitivity showed significant time-serial changes throughout the first week and month post-surgery in the operated eyes (*P* = 0.005, *P* = 0.045, and *P* = 0.043, respectively, Table 3). For untreated fellow eyes, the overall change of corneal erosions and Sjogren’s International Collaborative Clinical Alliance (SICCA) ocular surface staining (OSS) scores were significant (*P* = 0.012 and *P* = 0.010, respectively, Table 3).

**Table 3:** The repeated measures analysis of variance (RMANOVA) of the time-serial changes of various parameters for dry eye disease in both the operated eyes and the untreated fellow eyes within 1 month after cataract surgery.

RMANOVA		Group					
		Operated eye			Fellow eye		
Source	Parameters	df	F	*P* value	df	F	*P* value
Time	MG expressibility (Gr)	2	0.426	0.656	2	0.378	0.688
	Meibum quality (Gr)	2	6.078	0.005*	2	0.756	0.476
	Schirmer I without anesthesia (mm)	2	3.329	0.045*	2	1.203	0.309
	Corneal sensitivity (cm)	2	3.287	0.043*	2	0.010	0.990
	Tear BUT (sec)	2	0.114	0.893	2	0.085	0.919
	Corneal erosion score (NEI)	2	1.586	0.216	1.423	6.017	0.012*
	OSS score (SICCA)	2	2.186	0.124	1.262	5.107	0.010*

*MG* meibomian gland, *Gr* grade, *BUT* break-up time, *NEI* National eye Institute/Industry, *OSS* ocular staining score, *SICCA* Sjogren’s International Collaborative Clinical Alliance.

*P < 0.05.

**Figure 2:**
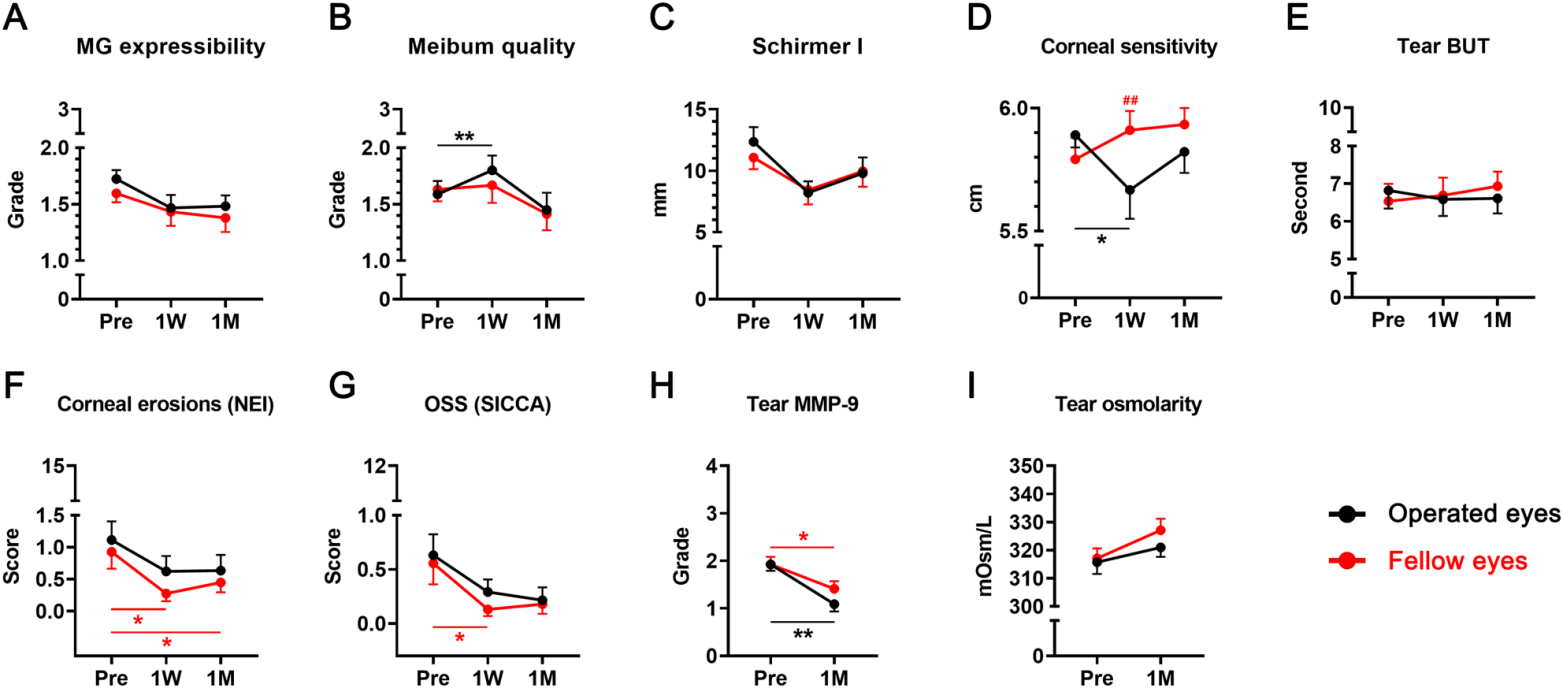
The short-term time-serial changes of ocular surface parameters before and after cataract surgery for 1 month. The operated eyes showed an increase in meibum quality grade and a decrease in corneal sensation at 1 week. On the other hand, the untreated fellow eyes displayed decrease of corneal erosion and OSS scores after surgery. Tear MMP-9 expression decreased in both eyes at 1 month after surgery. (**A**–**G**) *P < 0.05 and **P < 0.01 (One-way repeated measure analysis of variance analysis followed by Bonferroni’s *post-hoc*). (**D**) ^##^*P* < 0.01 (Mann–Whitney *U* test between groups at 1 week). (**H**, **I**) *P < 0.05 and **P < 0.01 (paired t-test). *W* week, *M* month, *MG* meibomian gland, *BUT* break-up time, *OSS* ocular staining score, *NEI* National Eye Institute/Industry, *SICCA* Sjogren’s International Collaborative Clinical Alliance, *MMP-9* matrix metalloproteinase-9.

Figure 2 illustrates the derailed time-serial changes of all DED parameters and MGD severity grades. Meibum quality grade increased significantly at 1 week in the operated eyes (*P* = 0.003, Fig. 2B). Although corneal sensitivity did not change in the fellow eyes, it decreased significantly in the operated eyes at 1 week (*P* = 0.012, Fig. 2D). Moreover, the difference in corneal sensitivity between the two groups was statistically significant at 1 week (*P* = 0.001, Fig. 2D). Although the scores of corneal erosions and SICCA OSS did not change postoperatively in operated eyes, corneal erosions improved both at 1 week and 1 month after surgery in the untreated fellow eyes (*P* = 0.023 and *P* = 0.023, respectively, Fig. 2F). The SICCA OSS scores also improved at 1 week in fellow eyes (*P* = 0.008, Fig. 2G). The tear matrix metalloproteinase (MMP)-9 grade decreased in both operated eyes and untreated fellow eyes after a month from surgery (*P* = 0.002 and *P* = 0.017, respectively, Fig. 2H).

Among all parameters, only meibum quality grade and corneal sensitivity showed different time-serial change patterns between the two groups (Table 4).

**Table 4:** The comparison of time-serial changes of various parameters for dry eye disease between in the operated eyes and untreated fellow eyes within 1 month after cataract surgery.

RMANOVA		df	F	*P* value
Source	Parameters			
Time × Group	MG expressibility (Gr)	2	0.598	0.555
	Meibum quality (Gr)	2	3.656	0.035*
	Schirmer I without anesthesia (mm)	1.596	2.777	0.086
	Corneal sensitivity (cm)	2	3.482	0.036*
	Tear BUT (sec)	2	0.067	0.935
	Corneal erosion score (NEI)	2	2.066	0.139
	OSS score (SICCA)	1.473	2.225	0.136

*MG* meibomian gland, *Gr* grade, *BUT* break-up time, *NEI* National Eye Institute/Industry, *OSS* ocular staining score, *SICCA* Sjogren’s International Collaborative Clinical Alliance.

*P < 0.05.

## Discussion

Although several reports have described newly occurred or aggravated DED after cataract surgery, our previous study emphasized the effect of active postoperative anti-inflammatory treatment, including the use of topical NSAIDs and steroid eyedrops^28^. This treatment was found to improve pre-existing ocular discomfort and decrease the amount of tear MMP-9 after cataract surgery. Bilateral ocular surface changes have been recently highlighted in cases of unilateral corneal infection, viral reactivation of herpes simplex virus and varicella-zoster virus, and corneal nerve injury^23–25,29–31^. Furthermore, it is well known that cataract surgery can induce inflammatory reactions on the ocular surface for various reasons^5,32^. Based on these findings, we hypothesized that the ocular surface parameters in the untreated fellow eye might improve after the short-term administration of anti-inflammatory treatment in eyes with cataract surgery. In this study, we evaluated the serial changes in bilateral ocular surface parameters before and after cataract surgery in patients with preoperative ocular surface discomfort. Interestingly, the untreated fellow eye showed a significant decrease in ocular surface erosions and tear MMP-9 expression, along with an overall decrease in OSDI scores after surgery.

Contrary to several studies that have reported worsening of dry eye after cataract surgery^33,34^, this study showed a significant decrease in OSDI scores after surgery. According to a recent study, changes in subjective symptoms, tear film break-up time (BUT), and corneal staining patterns after cataract surgery varied depending on the presence or absence of pre-existing dry eye^35^. The study showed that subjective symptoms of DED improved after surgery in patients with preexisting dry eye, whereas in patients without dry eye, corneal staining increased and tear film breakup time decreased. These two outcomes were maintained at the same level as before surgery without any worsening in DED patients^35^. In the present study, 91.2% of the subjects had preexisting dry eye according to OSDI scores, and among those with dry eye, 61.3% had scores of 33 or higher, indicating a severe type of dry eye and. The high proportion of subjects with dry eye in this study cohort, similar to the previous study^28,35^, may explain the similar results.

There are several points to consider regarding the ocular surface changes after cataract surgery. In fact, it is plausible that the meibum quality and corneal sensation decreased in operated eyes 1 week after surgery due to direct corneal nerve transection and temporary lid hygiene issues. In addition, the significant decrease in tear MMP-9 expression in operated eyes after 1 month is likely due to the anti-inflammatory effect of postoperative NSAID eye drops and high-potency steroid eye drops. However, what is surprising is the improvement of ocular surface erosions and tear MMP-9 expression in untreated fellow eyes, despite undergoing anti-inflammatory treatment only in operated eyes.

Despite the improvement in surface erosions and tear MMP-9 expression in both eyes, the OSDI scores did not correlate with any individual clinical parameters before and after surgery, which was unexpected. Given the multifactorial nature of DED, we find it challenging to identify a single culprit DED parameter that explains changes in OSDI scores. This hypothesis gains support from significant inter-parameter correlations observed between MG expressibility and meibum quality grades, MG expressibility grades and lacrimal secretion by Schirmer I test, lacrimal secretion and corneal erosion scores, tear BUT and corneal erosion scores, corneal erosion scores and SICCA OSS, and meibum quality grades and tear MMP-9 grades (Supplementary Table 1).

The OSDI score essentially reflects integrated symptoms from both eyes. Despite enrolling participants for unilateral cataract surgery in this study, we hypothesized that the overall improvement in OSDI scores after cataract surgery might be attributed to a bilateral decrease in MMP-9 levels in tears and contralateral surface erosion staining scores. Similar to other well-known questionnaires such as NEI-VFQ-25, the Standard Patient Evaluation of Eye Dryness Questionnaire, and the Dry Eye Questionnaire, the OSDI scoring system cannot discriminate symptoms between each eye. This limitation restricts our ability to interpret which eye contributed to the subjective improvement after surgery. Additionally, a follow-up study is required to identify which improvements among the OSDI questions may contribute to the overall improvement in the OSDI score after cataract surgery with short-term administration of anti-inflammatory eye drops.

Previous studies have demonstrated a significant decrease in corneal sensitivity around the incision site in the first week after cataract surgery, which gradually recovers over time^36,37^, as seen in this study. It is known that damage to sensory nerves can cause neurogenic inflammation^19^, and recent studies have shown that CD4^+^CD69^hi^ lymphocytes transiently increase on the ocular surface in the second week after injury in a mouse model with circular corneal nerve transection^26^. Based on these results, it is possible that transient neurogenic inflammation may occur in the early stages after cataract surgery. However, MMP-9 expression even decreased 1 month after surgery in this study, possibly because the examination only focused on MMP-9 expression at 1 month after surgery. Therefore, the inflammation induced by nerve damage at 1 week may not have been reflected in the present study. Alternatively, MMP-9 expression due to minimal corneal nerve damage with a 2.75 mm keratome may be at a mild level that can be easily overcome by administering anti-inflammatory eye drops after surgery.

The reason why the ocular surface parameters of the fellow eye improved with contralateral eye treatment is unclear and cannot be easily identified. However, one possible explanation is the bilateral immunopathogenic induction of dry eye. As we known, desiccation can activate antigen-presenting cells in the cornea, which then migrate to regional lymph nodes to expand type 17 helper T cells and antagonize regulatory T cells bilaterally^25^. According to this immunopathogenesis of dry eye, effector immune cells such as type 17 helper T cells may cross to the contralateral corneal surface through systemic circulation to induce intermittent inflammation accompanied by MMP-9 expression in tears. We believe that both eyes were immunogenically linked to express dry eye before cataract surgery in our cohort. If the immunogenic stimulus on the ocular surface in operated eyes were controlled and attenuated by postoperative anti-inflammatory eye drops, the ongoing effector immune cells that existed before surgery might have also been controlled after the surgery. We suggest that such a contralateral change could have been occurred because the baseline corneal erosion scores and SICCA OSS scores were very low. In addition, the significant but small decrease of tear MMP-9 grade in the fellow eyes also suggests that the contralateral anti-inflammatory effect in this study was not strong. In summary, it is difficult to conclude that unilateral anti-inflammatory treatment would be always able to control the opposite eye as well. We believe that it is just something like a simple surprise gift after surgery to attenuate the pre-existing subjective ocular discomfort. Alternatively, patients may have naturally mitigated potential aggravating factors for DED, such as screen time, after the cataract surgery. However, it is crucial to acknowledge that confounding factors affecting the postoperative improvement of DED severity cannot be completely ruled out in a retrospective design. Furthermore, to support the inter-eye immunological mechanism proposed for the improvement of DED in the fellow eye, as demonstrated in this study, a comparison with a control group in a prospective future study, where anti-inflammatory eye drops are instilled in both eyes after unilateral cataract surgery, would be helpful.

This study has several limitations. It was a retrospective cohort study based on medical records, and our institution did not perform tear MMP-9 and tear osmolarity measurements during the first weeks after cataract surgery as part of our routine clinical protocol. Although all subjects complained of ocular discomfort and most of them were considered to have DED according to OSDI scores, the severity of DED and subjective discomfort was not homogenous before surgery. Moreover, evaluating short-term serial changes in tear MMP-9 expression and tear osmolarity is needed to analyze initial inflammatory changes that are more dependent on cataract surgery itself. Nevertheless, our results suggest the possible improvement of the opposite eye along with the subjective improvement after cataract surgery. Additionally, the results may aid clinicians in explaining the possible decrease of ocular discomfort by postoperative medical treatment in DED patients who express their symptoms more severely than their corneal and conjunctival erosions.

In conclusion, the short-term anti-inflammatory treatment using NSAID and steroid eye drops in an eye with cataract surgery may improve the objective ocular surface parameters in an untreated fellow eye postoperatively. Furthermore, this might contribute to the overall subjective improvement of DED in patients with pre-existing ocular surface discomfort.

## Methods

This study was a retrospective short-term longitudinal cohort study. The whole process properly followed the tenets of the Declaration of Helsinki. The research protocol received approval from the Chung-Ang University Hospital Institutional Review Board (IRB), and considering the retrospective nature of the study design, the requirement for informed consent was waived by the IRB (Approval No. 2006-026-19320).

### Subjects

We identified 57 consecutive individuals who underwent unilateral cataract surgery in our institute between October 2019 and September 2020 and had preoperative ocular symptoms of ocular discomfort, such as dryness, foreign body sensation, burning, increased sensitivity to light, sensation of pressure, and frequent blinking and whose medical records of examinations for clinical parameters for DED and MGD were documented in medical charts. We excluded subjects with a systemic immunologic disease including Sjogren’s syndrome and allergic disease, those with pterygium, those under systemic immune-related treatments or topical administration of anti-inflammatory eye drops, autologous serum eye drops or anti-glaucomatous eye drops, those who had worn contact lens within the previous 3 months, those who had undergone ocular surgery within the previous 6 months, and those who performed interventional MGD treatment including intense pulsed light treatment, thermal pulsation treatment and meibomian gland expression within previous 6 months. However, we allowed the enrollment of subjects who had intermittently used artificial tears when experiencing ocular dryness before surgery.

### Study design

Our retrospective medical chart review study design was as follows:

1. Compare the demographics and preoperative ocular surface parameters of DED and MGD in an operated eye and in a fellow eye.
2. Analyze time-serial changes of DED parameters and MGD severity grades in each group.
3. Compare time-serial changes of DED parameters and MGD severity grades between the two groups.

### Cataract surgery and postoperative use of eye drops

The surgery was performed by the same surgeon under topical anesthesia. In all subjects, a 2.75 mm wide incision was made on the temporal periphery of the transparent cornea. Phacoemulsification (Centurion Vision System; Alcon Laboratories, Inc., Fort Worth, TX, USA) and posterior chamber intraocular lens implantation were performed, and the incision site was left unsutured. After the surgery, the patient was prescribed moxifloxacin eye drops (Vigamox; Alcon Laboratories) three times a day, preservative-free 1% prednisolone acetate eye drops (Predbell; Chong Kun Dang Holdings Corp., Seoul, Korea) four times a day, and preservative-free 0.1% bromfenac sodium hydrate eye drops (Bronuck; Taejoon Pharm., Seoul, Korea) twice a day for 1 month only in an operated eye. No other eye drops, including artificial tears, were used during the first month after the surgery.

### OSDI questionnaire

We used the OSDI questionnaire to assess the subjective ocular symptoms of DED and their effect on vision-related function^38^. The survey was targeted for a period of 1 week before the day of the survey and was composed of 3 subscales: ocular symptoms, vision-related daily function and environmental triggers. Patients answered twelve questions in total with scale from 0 to 4, 0 corresponding to “none” and 4 corresponding to “always”. Sum of scores multiplied by 25 were divided with the number of questions properly answered to calculated OSDI score^39^.

### DED parameters and MGD severity grades

Given that various pathogenic factors, including tear deficiency, tear film instability, and tear hyperosmolarity-induced ocular surface inflammation, are involved in DED^40^, and considering that MGD is highly prevalent after cataract surgery^8^, we collected the relevant multiple parameters to evaluate DED and MGD in this study. Specifically, tear MMP-9 was performed to reflect ocular surface inflammation indirectly, tear BUT was estimated to evaluate tear film instability, and the Schirmer I test was conducted to verify lacrimal insufficiency.

As a part of the assessment of DED parameters, we evaluated several clinical parameters, including corneal sensitivity scores, tear osmolarity (Tosm), tear MMP-9 severity grades, tear secretion with Schirmer I without anesthesia, tear BUT, SICCA OSS, corneal erosion scores, MG expressibility, and quality of the secreted meibum. The examination was performed in the order listed above. Considering that tear osmolarity and tear MMP-9 estimation are easily and even extremely influenced by the instillation of a solution on the ocular surface, we first conducted invasive tests, including corneal sensitivity, Tosm, tear MMP-9, and the Schirmer I test. We waited at least 15 min before the subsequent tear BUT estimation to eliminate the reflex tearing effect induced by the invasive tests.

To assess corneal sensitivity, we used a Cochet-Bonnet esthesiometer (Luneau ophthalmology, Chartres Cedex, France). Starting from the longest length of 6 cm, the test was performed by decreasing the length of the monofilament that touches the center of the cornea by 0.5 cm until the patient first felt discomfort.

To measure Tosm, we used an I-PEN (I-MED Pharma Inc., Montreal, QC, Canada) soaked with tear at the lower conjunctival fornix and then assembled into an analyzer that displayed test results in digits.

We performed the tear MMP-9 test using InflammaDry (Quidel, San Diego, CA, USA), following the instructions provided in the product documentation^41^. We used a sterile sample collector to dab multiple areas along the lower palpebral conjunctiva to collect tear fluid, which was then assembled into the immunoassay test cassette. The intensity of the red line in a readout window was verified after 20 s of activation in buffer solution. We analyzed the MMP-9 assay using a 5-scale grading method. The grading system involved grading the depth of the red readout band of the MMP-9 on-site inspection, with grade 0 indicating a negative result, grade 1 indicating a trace result, grade 2 indicating a weak positive result, grade 3 indicating a positive result, and grade 4 indicating a strong positive result, based on previously established standard photographs^42^.

To assess tear secretion, we conducted the Schirmer I test without anesthesia. This approach was chosen to better reflect the function of the lacrimal functional unit after cataract surgery, where corneal nerves are inevitably injured during the corneal incision. Schirmer standard strip (Eagle Vision, Memphis, TN, USA) was placed on the outer 1/3 point of the lower conjunctival fornix and allowing tear fluid to be absorbed for 5 min without using any analgesic eyedrops.

To measure tear BUT, we followed established procedures^43^ and conducted the measurement more than 15 min after the Schirmer I test. A drop of normal saline was placed on a strip of paper coated with fluorescein dye (Haag-Streit International, Koniz, Switzerland) and then shaken off. The strip was gently applied to the lower lid margin to stain the tear film, and the time when the first tear film break was observed under a cobalt blue filter after the last blink was considered the BUT. We repeated the measurement three times using a stopwatch and used the average value. Afterward, we evaluated the ocular staining score by examining each eye with a slit-lamp under a yellow filter after fluorescein instillation^44^.

The OSS score according to the SICCA scoring system^45^ and corneal erosions score according to the National Eye Institute/Industry (NEI) scoring sytem^46^ were obtained using established standards.

To evaluate MGD, we used two methods: assessing MG expressibility of five glands in the central upper lid and the quality of secreted meibum. The MG expressibility of meibum from five glands was graded from 0 to 3, with 0 indicating all glands expressible, 1 indicating 3–4 glands expressible, 2 indicating 1–2 glands expressible, and 3 indicating no glands expressible. The quality of meibum was also graded from 0 to 3, with each score corresponding to clear, cloudy, cloudy particulate fluid, and toothpaste-like consistency according to previously established benchmarks^47^. All DED clinical parameters were measured and evaluated consistently by a single experienced researcher (K.W.K).

### Statistical analysis

Prism software (GraphPad, La Jolla, CA, USA) and SPSS software (Chicago, IL, USA) was used for statistical tests. To analyze the trends in changes in serial grades for MG expressibility and meibum quality, lacrimal secretion by the Schirmer I test, threshold of corneal sensitivity, tear BUT, corneal erosion scores, and SICCA OSS over 1 month, a one-way repeated measures analysis of variance (RMANOVA) was performed. Bonferroni’s *post-hoc* test was conducted to verify the differences between each time-point in each group. To analyze the differences in Tosm and tear MMP-9 grade between before and 1 month after surgery, a paired *t*-test was employed. To determine if there were any differences in the trend changes between the two groups, a two-way RMANOVA was conducted. The difference in variables between the two groups at a particular time-point was evaluated using either the parametric Student’s *t*-test or the non-parametric Mann–Whitney *U* test. This assessment was conducted after checking the homogeneity of variance using a Levene test. The correlation between DED parameters and MGD severity grades was performed using Pearson’s correlation analysis. The data sets were expressed as an average and standard deviations ( ±), and all statistical analyses were considered significant at *P* < 0.05.

### Supplementary Information

Supplementary Table 1.

## Data Availability

The datasets generated and analyzed in the current study are available from the corresponding author on reasonable request.
