# Supplementary material for: Ocular surface parameter changes in the untreated fellow eye after unilateral cataract surgery with short-term administration of anti-inflammatory eye drops
Source: Sci Rep. 2024 Jan 11;14:1080. doi: 10.1038/s41598-024-51764-7 (PMC10784480; doi:10.1038/s41598-024-51764-7)
Supplement: Supplementary file 1 — Supplementary Table 1. [file 41598_2024_51764_MOESM1_ESM.pdf]

Supplementary Table 1. The correlation analysis between individual dry eye disease parameters and meibomian gland dysfunction severity grades.

Vs.	MG expressibility (Gr)	Meibum quality (Gr)	Schirmer I without anesthesia (mm)	Corneal sensitivity (cm)	Tear BUT (sec)	Corneal erosion score (NEI)	OSS score (SICCA)	Tear MMP-9 (Gr)	Tear osmolarity (mOsm/L)
**MG expressibility (Gr)**	–	*R*=0.507 *P*<0.0001*	*R*=0.167 *P*=0.024*	*R*=-0.060 *P*=0.406	*R*=-0.096 *P*=0.244	*R*=-0.010 *P*=0.906	*R*=-0.035 *P*=0.666	*R*=0.225 *P*=0.009*	*R*=0.055 *P*=0.582
**Meibum quality (Gr)**	–	–	*R*=0.025 *P*=0.733	*R*=-0.114 *P*=0.117	*R*=-0.027 *P*=0.743	*R*=0.150 *P*=0.069	*R*=0.066 *P*=0.417	*R*=0.175 *P*=0.045*	*R*=0.008 *P*=0.938
**Schirmer I without anesthesia (mm)**	–	–	–	*R*=-0.117 *P*=0.084	*R*=-0.018 *P*=0.819	*R*=-0.160 *P*=0.040*	*R*=-0.142 *P*=0.070	*R*=0.071 *P*=0.384	*R*=0.010 *P*=0.914
**Corneal sensitivity (cm)**	–	–	–	–	*R*=0.015 *P*=0.850	*R*=-0.138 *P*=0.077	*R*=-0.092 *P*=0.237	*R*=-0.092 *P*=0.257	*R*=0.010 *P*=0.924
**Tear BUT (sec)**	–	–	–	–	–	*R*=-0.201 *P*=0.011*	*R*=-0.204 *P*=0.009	*R*=-0.173 *P*=0.086	*R*=-0.155 *P*=0.133
**Corneal erosion score (NEI)**	–	–	–	–	–	–	*R*=0.756 *P*<0.0001*	*R*=0.142 *P*=0.164	*R*=0.025 *P*=0.804
**OSS score (SICCA)**	–	–	–	–	–	–	–	*R*=0.084 *P*=0.401	*R*=0.022 *P*=0.828
**Tear MMP-9 (Gr)**	–	–	–	–	–	–	–	–	*R*=-0.001 *P*=0.998
**Tear osmolarity (mOsm/L)**	–	–	–	–	–	–	–	–	–

MG, meibomian gland. Gr, grade. BUT, break-up time. NEI, National Eye Institute/Industry. OSS, ocular staining score. SICCA, Sjogren's International Collaborative Clinical Alliance. MMP-9, matrix metalloproteinase 9. *$P<0.05$ (Pearson's correlation analysis).
